# Need and challenges of palliative care in tribal people: a qualitative analysis

**DOI:** 10.1186/s12904-025-01711-8

**Published:** 2025-03-20

**Authors:** S. B. Keerthana, A. Kubendran

**Affiliations:** Department of Social Sciences, School of Social Sciences and Languages, Vellore Institute of Technology, Vellore, 632014 India

**Keywords:** Tribal people, Palliative care, Need, Challenges, Culture, Health, Wellbeing

## Abstract

**Background:**

Indigenous people are the most vulnerable and marginalised parts of society. Health services available to the tribal people are in developing face when compared to non-indigenous people. The situation with palliative care services exhibits a similar discrepancy. This study aims to explore the challenges and needs of palliative care within the tribal communities of Kerala.

**Methods:**

The study followed a case study method conducted among the Paniya tribal community in Kambhatti, Maani, and Ozhakodi settlements of the Wayanad district, Kerala. The researchers used semi-structured interviews and observation as data collection methods. The data were collected from tribal people, ASHA workers, and promoters. Tribal people older than 18 years are considered for this study. A six-step model of reflexive thematic analysis was used for analysis. Open coding, followed by axial coding, was used to analyse the data collected from the participants. The codes were combined to form themes.

**Results:**

A total of 12 participants were interviewed for the study, among them 8 participants were tribal people, 2 participants were promoters and 2 participants were ASHA workers. The majority of the tribal individuals live in joint families inside their settlement. Most of the participants were women, compared to men. Most of the women are employed through the Mahatma Gandhi National Rural Employment Guarantee Act. Nine major themes related to the challenges of palliative care were derived after the analysis are, lack of awareness, accessibility, financial issues, cultural beliefs and practice, fear and communication barriers, insufficient health care workforce, palliative care services, and health-related issues.

**Conclusions:**

This study highlights the significance of palliative care for tribal populations. There is an increased need for palliative care, accompanied by challenges with receiving it in a culturally acceptable and sensitive manner. Developing a hybrid healthcare approach that integrates both traditional and modern medications exclusively for tribes is essential. The tribal population requires support in accessing comprehensive palliative care services from various professionals. It is crucial to establish healthcare programs aimed at addressing the entire healthcare needs of the tribal populations.

## Background

Palliative care is holistic care provided to the patient and their family with a life-limiting illness [1]. This treatment may enhance the patient’s and their family’s quality of life physically, psychologically, socially, and spiritually [2]. Palliative care is required for an estimated 56.8 million individuals annually, of whom 25.7 million are in their last year of life [3]. Only 14% of patients who need palliative care are receiving it around the world, and 98% of patients in need of palliative care are living in low-and middle-income countries (LMICs) [3]. According to the Quality of Death Index, India is placed 67th among the 80 surveyed nations, indicating that the quality of dying is less in India and it might be enhanced via the effective implementation of palliative care nationally [4]. Arokiasamy (2018) states that the prevalence of chronic illnesses is also significantly high in India when compared to other countries [5]. The need for palliative care is far higher in rural communities, where its implementation is minimal compared to urban areas in India [6]. According to the study conducted by Kumar (2013), the number of people dying each year in India is more than 9.8 million and the number of people in need of palliative care will be 6 million [7]. This indicates an urgent need for palliative care in all parts of India.

Palliative care was established in India in the 1980s. However, it is not widely available there yet, and many challenges remain, such as geographical diversity, poverty, and high population density [8]. Gujarat was first chosen for palliative care; however, places such as Kerala, Maharashtra, Karnataka, and Delhi have seen a rise in these initiatives [8]. Kerala is one of the states that has developed an effective palliative care strategy that has been adopted both in India and worldwide [8]. Kerala announced its palliative care policy in 2008, which was subsequently changed in 2019 after extensive revision [3]. 

## Palliative care in tribal settings: Kerala

The palliative care policy had an impact on all aspects of healthcare. The revised palliative care policy of Kerala emphasises support for highly vulnerable groups, including scheduled tribes residing in isolated regions. The term “scheduled tribe” refers to indigenous communities in India. The Indian government acknowledged them as a socially and economically disadvantaged population. The Kerala model of palliative care stresses the key points of ‘Inclusion and accessibility, community-based approach, Integration with traditional medicine, and training and education’ [3]. It emphasises the training and education of local volunteers and healthcare workers to deliver culturally sensitive palliative care with traditional medicines such as Siddha and Ayurveda [3]. According to the World Health Organisation (WHO), Palliative care should be provided following the principles of universal health coverage, which state that everyone should have access to a nationally determined set of basic health services, regardless of income, disease type, or age [9]. 

There were around 75 groupings of tribes found in India, 17 states, and one union territory they come under particularly vulnerable tribes [10]. Since 1961, the population of Indian Scheduled Tribes (STs) has undergone growth, at a rate of 21.3% in the 2011 census [11]. Kerala also has a separate tribal community; most of them live in the western ghats bordering Tamil Nadu and Karnataka. The tribes living in various regions of the state possess a wealth of culture. The largest tribe in Kerala is the Paniya [12]. The 2011 census data states that there are 88,450 Paniyas or 18.24% of the State’s total tribal population [13]. Their way of existence is shaped by the surrounding natural environment. The Paniya tribes are those who were historically subjected to severe enslavement by the upper caste and continue to be greatly underprivileged and deprived [14]. The Paniyas place minimal emphasis on education, and this low literacy rate is the fundamental reason for their socio-economic challenges [15]. The incidence of illnesses and the poverty rate among the Paniya tribe are significantly higher in comparison to other groups [14]. The Paniyas exhibit a markedly low rate of healthcare usage, with 30% of the Paniya population not utilising the advantage of healthcare facilities. Special tribal schemes exist exclusively for Paniyas; nonetheless, the utilisation rate is significantly lower compared to other tribes [14, 16].

Tribal populations constitute the most poor and neglected members of our nation [17]. The health status of tribal members differs from that of non-tribal members [17, 18]. They only had restricted access to healthcare services [19]. According to the reports, the most prevalent medical conditions in a tribal community include cancer, hypertension, stroke, anaemia, pregnancy-related problems, early birth, malnutrition, substance abuse-related issues and anaemia [17]. An individual with these non-communicable diseases will experience emotional, social, and spiritual challenges in addition to physical problems. Palliative care is important in this situation to enhance the individual’s overall quality of life. Culturally sensitive quality treatment is essential for addressing these issues in tribal communities. The palliative care policy of Kerala explicitly outlines its advantages for tribes; yet, its execution remains uncertain. Even though most non-tribal persons now have access to palliative care, the tribal population still does not receive it properly [20]. A significant gap exists in the implementation of palliative care in Kerala, particularly in rural regions [21]. A community-based approach to palliative care, as outlined in the policy, should be actively implemented in a tribal settlement. It is essential for improving the quality of life for the tribal community in a respectful and responsive manner. There is a lack of research regarding tribal palliative care, particularly in Kerala. The present study aims to comprehend the needs and challenges of palliative care within the tribes. Since they are a part of our world as well, we must uphold equity in everything.

### Methods

The study explores the needs and challenges of palliative care among Paniya tribes and tribal attitudes regarding the health care services provided in Kerala’s Wayanad region. The Paniya tribe is the largest scheduled tribe in Kerala, primarily located in the district of Wayanad [22]. Paniya tribes are predominantly agricultural labourers [23]. The researchers visited the tribal habitat in July 2024 with the help of promoters within the respective tribes. Paniya Colonies, namely Kambhatti, Maani, and Ozhakodi, are mainly visited for data collection.

The present study was qualitative and used a case study research design. According to Yin (2009), A case study is empirical research that examines a phenomenon in its real-life context [24]. The participants were tribal people from the Paniya colony and Promoters and ASHA (Accredited Social Health Activists) workers. The researcher employed a collaborative method to select participants, which was crucial in ensuring that the community’s diverse perspectives were represented. Table 1 lists the inclusion and exclusion criteria. After receiving ethical approval from the institutional ethics committee, the researchers visited the field to gather data. The researchers interviewed eight tribal people two tribal promoters and two ASHA workers. Respondents were identified at random with the help of promoters based on language preference (Malayalam) and the data was gathered from the respondents who expressed their willingness to participate in the research study. The data collection process continued until data saturation was reached.

**Table 1:** Inclusion and exclusion criteria

Inclusion Criteria	Exclusion Criteria
Paniya tribal People from Kambhatti, Maani, and Ozhakodi colonies	Paniya tribal people from other parts of Kerala
Paniya tribal people from Wayanad district	Tribal people with mental health impairments, or any other disabilities
ASHA workers and Promoters in these selected tribal settlements	Children and adolescents
Tribal people older than 18 years are considered	The tribal people who are not willing to participate

The researchers collected data using semi-structured interviews and observations. Both groups of participants were interviewed using a predefined checklist (Table 2). The interview questions are open-ended and repeatedly updated. The interview guide was used to cover the target respondents, such as tribal people, promoters, and ASHA workers. The researchers conducted non-participant observation during data collection. Observations are recorded as notes by the researcher during the field visit. The researchers acknowledged that field observation notes may contain researchers’ preconceptions; to mitigate this, field notes were frequently reviewed.

**Table 2:** Semi-Structured interview guide

**1**	Socio-demographic data Age Gender Occupation Marital status Family type
**2**	What is their awareness about health care services?
**3**	What is their awareness of palliative care?
**4**	What are the health conditions commonly seen in your settlement and the need for palliative care?
**5**	How do their traditional beliefs and practices influence the perception of the illness and care?
	How they are dealing with chronic illnesses in their settlement?
**6**	What are the challenges faced by tribal peoples in terms of health care?
**7**	What are the available healthcare services and programs in this settlement?
**8**	Does the available healthcare services are getting the tribal people properly?
**9**	Health status of tribal people
**10**	Is there any educational program conducted to increase their awareness among tribal people?
**11**	Challenges of proper implementation of health care services.

All communications with participants are only carried out with their consent. A written consent was obtained from each of the participants before the interview. Confidentiality of the provided information has been preserved in every possible way. The researchers submitted the study proposal to the director of the tribal development department of Kerala in February 2024 and received approval to carry out the study in July 2024. Data collection was started only after getting permission to visit the settlement from the director of the tribal development department of Kerala. The researchers followed all protocols specified in the approval letter throughout the data collection process. The researchers also had a comprehensive understanding of the tribal community and their cultural beliefs before visiting the community, which helped to prioritise cultural sensitivity and reduce bias. The collected information was recorded with the help of a digital audio recording device and observation notes. The data collection process was completed in one or more sittings. The average duration of each interview was 90 min. The Paniya tribes frequently speak a language that is predominantly connected to Malayalam, with a mix of Tamil and Tulu. The native term used by the tribal respondents in interviews was elucidated with the assistance of promoters during the data collection process. In such cases, the researchers employed Malayalam equivalents when they were well-understood and accepted within the community. The researchers engaged in ongoing critical reflections to reduce our own biases and assumptions. Data were translated from Malayalam to English, and the transcribed data was checked by a professor who had proficiency in both languages. A six-step model of reflexive thematic analysis (RTA) proposed by Braun and Clarke was used for analysis [25]. The RTA highlights the importance of researchers’ reflexivity and maintains flexibility regarding theoretical independence. This technique was structured according to constructivist epistemology, and the inductive analysis entailed an open, flexible, and iterative process. Each researcher carefully read and re-examined the data to establish familiarity. The researchers separately coded the data using open coding followed by axial coding. The codes were compared and discussed by both researchers. Themes were formed from the combination of the codes.

One of the researchers in this team had one year of experience working as a psychologist in a palliative care setting in Kerala, this enhanced their awareness of human suffering and the emotional difficulties of a person with life-limiting illnesses. This experience has cultivated deep empathy and enhanced the researcher’s ability to communicate with the participants. Another researcher had experience working with one of the tribal groups in Tamil Nadu. This background greatly enhanced our comprehension of the socio-cultural circumstances influencing individuals’ lives and behaviours. Each tribe is unique based on their way of life and cultural practices. However, there is a chance of preconceptions during the research process. The opportunity for interviewer bias was significant here. The interview process was conducted within their tribal settlement, and their own houses to ensure the comfort of the tribal individuals and minimise bias. Reflexive practices were facilitated by field observation notes and reflexive journaling following the interviews. The researchers also engaged in collaborative reflective discussions with one another. Recall bias was avoided through accurate data recording. Further exploration during interviews was facilitated by the additional insight into responses that were derived from personal experiences. The internal validity of the obtained data was established through member checks with four participants in the study: three tribal individuals and one ASHA worker. The researcher elucidated the transcript to them to authenticate the information they shared and verify their original statements.

## Result

The Paniya tribes comprise most of the population, in the Wayanad district [26]. Each tribal settlement (Hamlet) contained between ten and fifteen families [22]. A significant proportion of tribal males work in agriculture and construction, while the majority of women seek jobs through the Mahatma Gandhi National Rural Employment Guarantee Act (MGNREGA) [27]. A total of 12 participants were interviewed in the study, among them 8 participants were tribal people, 2 participants were promoters and 2 participants were ASHA workers. In comparison to men, women are the majority of participants. Most tribal people live in joint families. A joint family is a household with three or more generations cohabiting. Other details of the participants are provided in Table 3. 9 primary themes emerged from the analysis of the interview transcript. Participant’s quotes connecting the themes and subthemes are provided in Table 4.

**Table 3:** Sociodemographic detail of the participants

Participant	Age	Gender	Occupation	Marital status	Family type
P01	54	F	MGNREGA	Married	Joint family
P02	63	M	Nil	Married	Joint family
P03	32	F	MGNREGA	Married	Joint family
P04	26	F	Nil	Married	Nuclear family
P05	28	M	Construction work	Married	Joint family
P06	56	F	MGNREGA	Married	Nuclear family
P07	24	F	Nil	Married	Joint family
P08	20	F	Nil	Unmarried	Joint family
Promoter-1	24	M	Student	Unmarried	Joint family
Promoter-2	53	M	Farming	Married	Nuclear family
ASHA worker-1	34	F	ASHA worker	Married	Nuclear family
ASHA worker-2	29	F	ASHA worker	Married	Nuclear family

**Table 4:** Reflexive thematic analysis: major themes & Sub-themes

Verbatim of the participants	Initial codes	Subthemes	Meaning of subthemes	Major themes
*‘We are not conscious of these changes in our body. For example*,* older people may experience low blood pressure*,* causing them to faint frequently. We don’t usually consult them in hospitals*,* we will go there in the last stage. We only got to know about the problem after reaching the hospital.’ - P02* *‘I don’t have that much idea about my illness. I’m daily taking the tablets prescribed by the doctor. I will tell the ASHA worker*,* if the tablet runs out*,* and she will bring me some more.’- P03* *‘There is one person near my house with some serious disease named cancer*,* he was also bedridden*,* we do not interact with that family that much*,* my daughter’s children are there at my house*,* what if they get this disease?’- P01* *‘I don’t have any idea about the medical schemes existing for tribal people*,* we are not getting anything.’- P01* *‘A person has high blood sugar*,* which begins to harm their leg over time. He didn’t tell us anything*,* and it ended up getting complicated. They are not aware of the seriousness of the disease conditions’ -ASHA worker* *‘First*,* they will try several Ayurvedic treatments; they will only inform me when the illness is really severe and cannot be under control.’-Promoter-1*	Unaware of bodily changes, unwellness to consult a doctor, lack of knowledge about their health issues, fear of diseases, lack of awareness about their rights, Ayurvedic medication	Health-related knowledge, Fear of disease transmission, Healthcare schemes, healthcare-seeking behaviour.	Health-related knowledge- lack of knowledge related to their health issues and symptoms. Fear of disease transmission- misconception about non-communicable diseases that will spread through interaction. Healthcare schemes- Lack of knowledge about the existing health care schemes for tribes. healthcare-seeking behaviour- poor understanding about when and where to seek help from medical professional.	Lack of awareness
*‘It will take a while to get there in the hospital because there aren’t many vehicle facilities in this area*,* therefore we want to wait for a long time to get a vehicle.’-P07* *‘If any medical emergency comes*,* we have to wait a lot to get an auto from this area to the hospital-P05* *‘The tribal settlements are difficult to access because they are in remote areas*,* most of the tribal settlements are in hilly areas Transport facilities are very less in the area’-Observation*	Geographical location of tribal settlement, lack of transport facilities,	Location of tribal settlement, transportation	Location of tribal settlement- tribal settlements are located in remote and hilly areas. Transportation- Lack of vehicle facilities in tribal areas.	Accessibility
*‘I’m the only one who makes money for my family and children; my husband doesn’t give me any money; he drinks every day with the money he gets from his job.’-P06* *‘Once tribal people’s jobs are over*,* the owner of the workplace offers them less money and more alcohol to make them consistent in the workplace. This is one among the elements causing their increasing alcohol usage and related problems among tribes.’- ASHA worker-2* *“I don’t have enough money to cover my hospital costs.” Before we have enough money because of visiting the forest areas*,* we will spend one to two weeks in the forest by gathering honey*,* coconuts*,* and other healing plants to sell at the store. We aren’t going into the forest anymore because we have other work to do. All the women here are looking for MGNREGA*,* while the men are working in jobs like construction and farming. So that we get a regular amount of money.’- P03* *‘The tribes are only getting very cheap wages for their hard work’- Promoter-2*	Financial strain on women, Alcohol dependency, lack of money to access healthcare, underpaid daily wages jobs.	Alcohol consumption, Underpaid Daily wages jobs	Alcohol consumption- Alcohol consumption after receiving payment from the job Underpaid Daily wages jobs- tribal people are receiving low payments even after doing hard work.	Financial Issues
*‘Ayurvedic medicines are part of our belief systems*,* this allopathy medication doesn’t have that much power to give relief’- P03* *‘Medications supplied by doctors are ineffective since diseases will recur once the advised dosage is completed.’- P04* *‘The tablets prescribed were temporarily giving relief*,* but Ayurvedic medicine was not like that if we are using Ayurvedic medicine it will completely cure the disease’- P03* *‘First*,* they will try several Ayurvedic treatments; they will only inform me when the illness is really severe and cannot be under control by their natural medicines.’-Promoter-1* *‘One child in this settlement is having a liver issue*,* and the child’s parents are not willing to go to the hospital for treatment*,* the parents are saying that allopathy treatment is complicated and the child will be hurt so we are not consulting the allopathic healthcare practitioner.’- P08* *‘Now also some of the people are doing rituals to get rid of the disease*,* recently one incident happened for a child. The child had a fever for long days. The family members didn’t report me about this and they started consulting a priest*,* Finally*,* we found that and admitted that child to the nearby government hospital.’- ASHA worker 1*	Trust in Ayurvedic medication, ineffective medicine, Preference for traditional medicine, fear of allopathy health care, cultural practices	Trust in Ayurvedic medication, Attitude towards allopathy, Cultural practices	Trust in Ayurvedic medication- Strong belief in ayurvedic treatment and medicines. Attitude towards allopathy- negative thoughts and feeling towards allopathic medicine. Cultural practices- rituals that exists in tribes for healing.	Cultural Belief and Practice
*‘Language-related issues are also somewhat common in our community; sometimes the medical camp’s physicians and nurses will not understand what we are talking about.’. -P04* *‘We lacked the confidence to engage with the other people living outside our colony; their attitude toward us is unsatisfactory’-P07* *‘Most of the tribal people are not ready to talk with the researcher and even they are not coming out of their huts’- Observation* *‘Fear of judgment and anxiety make most of them interact with outsiders hardly at all. They won’t directly interact with the medical professionals.’- ASHA worker*	Language-related issues among tribes, lack of confidence, lack of interaction with outsiders, fear of judgement.	Language barrier, Social withdrawal, Fear of judgement	Language barrier- difficulty in communication. Social withdrawal- Someone’s choice to avoid social interaction because of fear, anxiety, or lack of confidence. Fear of judgement- Anxiety or distress that others judge them negatively	Fear and Communication Barrier
*‘We are not getting other grains; rice is what we eat most of the time as the ration shop supplies only that mostly. ’- P04* *‘Every time*,* we have black tea and rice porridge. We don’t have enough money to buy fruits and meats.’-P05* *‘The women are unhealthy. Doctors assert that some minerals are lacking in these pregnant ladies*,* which is the reason they are not healthy enough to deliver a child.’- ASHA worker-1* *‘Everyone*,* but particularly women*,* are extremely thin and unhealthy*,* this is one of the reasons for the onset of diseases’- Observation*	Limited availability of required food, health issues due to lack of nutrients	Food availability constraints, Malnutrition	Food availability constraints- limited availability of diverse nutrients Malnutrition- Undernourishment in the community and associated health issues	Poverty
*‘The promoters won’t come regularly*,* sometimes they won’t pick up the call also’- P07* *‘Sometimes the assigned healthcare workers won’t pick up the call’- P01* *‘I did not get paid at the time of the delivery of my second child when I contacted the promoter about it*,* he said*,* We don’t have enough funds with us right now*,* I will discuss this with the authorities and call you back. After that*,* he didn’t get back to me.’- P07* *‘The doctor and nurse come to our settlement once every two months’- P02* *‘Medical camp is only held here once a month or once every two months*,* it is challenging to either monitor or follow-up our issues*,* and we are again consulting them in their next month visit.’- P04* *ASHA workers are not giving any medications; rather*,* we are buying them from the hospital.’- P04*	Lack of proper support from assigned healthcare workers, not getting government funds, inconsistent visits, inconsistent conduction of medical camps	Lack of support from healthcare workers, Inaccessibility of government funds, inconsistent medical follow-up	Lack of support from healthcare workers- inadequate assistance from assigned health care workers. Inaccessibility of government funds- Not receiving needed government funds to the tribes. inconsistent medical follow-up- inconsistent delivery of healthcare services for the tribes	Insufficient Healthcare Workforce
*‘Palliative care services are not available in our settlement’- P06* *‘Palliative care nurse is visiting one patient once a month*,* who is unable to get out of bed*,* they are not providing proper follow-up’- P07* *‘The palliative care nurses will visit the patients*,* who are unable to get out of bed; I will accompany them for assistance. They will visit once a week or twice.’- ASHA worker-1* *‘There are two to three stroke patients here in our settlement*,* palliative care services are provided to them once a month’- ASHA worker-2* *The visiting palliative care team only includes nurses and doctors. Other professionals are not visiting the settlement. -Observation*	Lack of proper palliative care services, inconsistent services, palliative care nurse, lack of multidisciplinary care	Lack of consistent palliative care services, inadequate follow-up, Lack of multidisciplinary care	Lack of consistent palliative care services- inconsistent delivery of palliative care services in tribal settlement inadequate follow-up- improper follow-up from palliative care team. Lack of multidisciplinary care- lack of psycho-social support from palliative care team.	Palliative Care Services
*‘My grandfather had jaundice*,* which caused him to eat everything without realizing he had the illness. His death was brought on by a delayed diagnosis.’- P08* *‘One person has been diagnosed with oral cancer in this settlement*,* that is mainly because of the uncontrolled tobacco use’-ASHA worker-1* *“Patients with cancers of the mouth*,* throat*,* and stomach are there in some settlements” -AHSA worker-1* *“5 Stroke patients are there because of tobacco use”-ASHA worker-1* *“Many of the patients have problems with their blood pressure*,* which can lead to stroke. All of this was brought on by an unhealthful diet.”-ASHA worker 2* *There is a lifestyle change happening among tribes from traditional to modern- observation.*	Uncontrolled tobacco use, substance use diseases, health issues due to unhealthy diet, lifestyle change	Alcohol and tobacco-induced diseases, Lifestyle diseases	Alcohol and tobacco-induced diseases- Health conditions develop due to uncontrolled alcohol and tobacco use. Lifestyle diseases- non-communicable diseases caused due to lifestyle change.	Health-related Issues

### Master theme 1: Lack of awareness

The tribal participants reported that the individuals remain mostly unaware of the physiological changes occurring in their bodies and whether the diseases with which they are diagnosed are communicable or non-communicable.

*‘We are not conscious of these changes in our body. For example*,* older people may experience low blood pressure*,* causing them to faint frequently. We don’t usually consult them in hospitals*,* we will go there in the last stage. We only got to know about the problem after reaching the hospital.’ - P02*.

*‘I don’t have that much idea about my illness. I’m daily taking the tablets prescribed by the doctor. I will tell the ASHA worker*,* if the tablet runs out*,* and she will bring me some more.’- P03*.

‘*There is one person near my house with some serious disease named cancer*,* he was also bedridden*,* we do not interact with that family that much*,* my daughter’s children are there at my house*,* what if they get this disease?’- P01*.

Healthcare schemes available to the tribal people are not reaching them properly and they are also not aware of the existing schemes exclusively for Paniya.

*‘I don’t have any idea about the medical schemes existing for tribal people*,* we are not getting anything.’- P01*.

Health-seeking behaviour is poor among tribes, as they do not communicate their issues.

*‘A person has high blood sugar*,* which begins to harm their leg over time. He didn’t tell us anything*,* and it ended up getting complicated. They are not aware of the seriousness of the disease conditions’ -ASHA worker*.

*‘First*,* they will try several Ayurvedic treatments; they will only inform me when the illness is really severe and cannot be under control.’-Promoter-1*.

### Major theme 2: Accessibility

Most tribal villages are situated in remote, hilly regions of Kerala, resulting in inadequate transportation to reach existing facilities. Vehicles and ambulances cannot access the tribal hamlet due to the lack of developed roads. Tribal people have to wait so long for transportation from their settlement to the town. This may be one reason for the inconsistent visits of healthcare professionals or the palliative care team to the settlement.

*‘It will take a while to get there in the hospital because there aren’t many vehicle facilities in this area*,* therefore we want to wait for a long time to get a vehicle.’-P07*.

*‘If any medical emergency comes*,* we have to wait a lot to get an auto from this area to the hospital-P05*.

*‘The tribal settlements are difficult to access because they are in remote areas*,* most of the tribal settlements are in hilly areas Transport facilities are very less in the area’-Observation*.

### Major theme 3: Financial issues

The interviewees often cited financial insufficiency as a significant issue during the interview. This is a primary factor for the limited accessibility of available resources. Many tribal women are engaged in MGNREGA, while males are involved in construction and agricultural labour. The majority of female participants reported that the consumption of alcohol was the primary cause of financial difficulties in their families.

*‘I’m the only one who makes money for my family and children; my husband doesn’t give me any money; he drinks every day with the money he gets from his job.’-P06*.

In many instances, the sole source of income for a household was the remuneration received from MGNREGA. Sometimes this money was not enough for them to meet their basic expenses and medical emergencies.

*“I don’t have enough money to cover my hospital costs.” Before we have enough money because of visiting the forest areas*,* we will spend one to two weeks in the forest by gathering honey*,* coconuts*,* and other healing plants to sell at the store. We aren’t going into the forest anymore because we have other work to do. All the women here are looking for MGNREGA*,* while the men are working in jobs like construction and farming. So that we get a regular amount of money.’- P03*.

The workplace owners are offering alcohol with daily wages to tribal people as an incentive for consistency at work. This is one of the reasons for increased health-related issues among tribes.

*‘Once tribal people’s jobs are over*,* the owner of the workplace offers them less money and more alcohol to make them consistent in the workplace. This is one among the elements causing their increasing alcohol usage and related problems among tribes.’- ASHA worker-2*.

*‘The tribes are only getting very cheap wages for their hard work’- Promoter-2*.

### Major theme 4: Cultural belief and practice

Most of the participants expressed their trust in Ayurvedic medication when compared to allopathy. A participant stated that allopathic medication was ineffective, as the condition would readily recur upon completion of the treatment. The tribal people mistrust the allopathic treatment procedure, believing it will harm the patient more than it will heal.

*‘Ayurvedic medicines are part of our belief systems*,* this allopathy medication doesn’t have that much power to give relief’- P03*.

*‘Medications supplied by doctors are ineffective since diseases will recur once the advised dosage is completed.’- P04*.

*‘The tablets prescribed were temporarily giving relief*,* but Ayurvedic medicine was not like that if we are using Ayurvedic medicine it will completely cure the disease’- P03*.

They will seek allopathic treatment only at the most advanced stage of the disease progress.

*‘First*,* they will try several Ayurvedic treatments; they will only inform me when the illness is really severe and cannot be under control by their natural medicines.’-Promoter-1*.

The attitude towards allopathic medication is different in tribal people.

*‘One child in this settlement is having a liver issue*,* and the child’s parents are not willing to go to the hospital for treatment*,* the parents are saying that allopathy treatment is complicated and the child will be hurt so we are not consulting the allopathic healthcare practitioner.’- P08*.

They are currently performing rituals to get cure from severe illnesses within their community. Some priests still exist in certain settlements to perform these rituals.

*‘Now also some of the people are doing rituals to get rid of the disease*,* recently one incident happened for a child. The child had a fever for long days. The family members didn’t report me about this and they started consulting a priest*,* Finally*,* we found that and admitted that child to the nearby government hospital.’- ASHA worker 1*.

### Major theme 5: Fear and communication barrier

Tribal people are less socialised when compared to non-tribal people. They live together in isolated places in a separate tribal settlement (Hamlet). Most of the participants are living as a joint family in their settlement. The participants are hesitant to speak with non-tribal people for fear of being judged. One of the tribal participants mentioned that the physicians in the medical camp don’t always understand some words in their language.

*‘Language-related issues are also somewhat common in our community; sometimes the medical camp’s physicians and nurses will not understand what we are talking about.’. -P04*.

*‘We lacked the confidence to engage with the other people living outside our colony; their attitude toward us is unsatisfactory’-P07*.

*Most of the tribal people are not ready to talk with the researcher and even they are not coming out of their huts- Observation*.

Their hesitation to engage with professionals is a contributing factor to their inadequate health-seeking behaviour.

*‘Fear of judgment and anxiety make most of them interact with outsiders hardly at all. They won’t directly interact with the medical professionals.’- ASHA worker*.

### Major theme 6: Poverty

Poverty has been a pervasive issue in every tribal community for an extended duration. Tribal people are getting food via ration cards at a subsidised rate. Nevertheless, the majority of tribal people reported receiving only rice grains from the ration shop.

*‘We are not getting other grains; rice is what we eat most of the time as the ration shop supplies only that mostly. ’- P04*.

*‘Every time*,* we have black tea and rice porridge. We don’t have enough money to buy fruits and meats.’-P05*.

Malnutrition is a common problem faced by tribal women and children. Pregnant ladies are lacking enough minerals, which is affecting their child’s health as well.

*‘The women are unhealthy. Doctors assert that some minerals are lacking in these pregnant ladies*,* which is the reason they are not healthy enough to deliver a child.’- ASHA worker-1*.

*‘Everyone*,* but particularly women*,* are extremely thin and unhealthy*,* this is one of the reasons for the onset of diseases’- Observation*.

### Major theme 7: Insufficient healthcare workforce

Some tribal people reported that the promoters and ASHA workers occasionally fail to respond to their emergencies.

*‘The promoters won’t come regularly*,* sometimes they won’t pick up the call also’- P07*.

*‘Sometimes the assigned healthcare workers won’t pick up the call’- P01*.

At certain points, the tribal people fail to get the funds allocated to them through certain government programs.

*‘I did not get paid at the time of the delivery of my second child when I contacted the promoter about it*,* he said*,* We don’t have enough funds with us right now*,* I will discuss this with the authorities and call you back. After that*,* he didn’t get back to me.’- P07*.

Medical camps are conducted bi-monthly from the nearby public health centres (PHC) in their settlements. Palliative care nurses also visit their settlement once a month; nevertheless, there is inadequate follow-up in these two instances. Consistency in medical care was found to be poor.

*‘The doctor and nurse come to our settlement once every two months’- P02*.

*‘Medical camp is only held here once a month or once every two months*,* it is challenging to either monitor or follow-up our issues*,* and we are again consulting them in their next month visit.’- P04*.

*ASHA workers are not giving any medications; rather*,* we are buying them from the hospital.’- P04*.

### Major theme 8: Palliative care services

Palliative care services are accessible to all individuals in Kerala; yet, there remains a disparity concerning tribal populations. The majority of tribal individuals are unaware of palliative care and indicated that they have not received any services from a palliative care team.

*‘Palliative care services are not available in our settlement’- P06*.

Some tribal respondents said that palliative care nurses visit their settlement once a month. Furthermore, they are not delivering consistent follow-ups. The palliative care team is not providing any psychosocial support to the tribal people.

*‘Palliative care nurse is visiting one patient once a month*,* who is unable to get out of bed*,* they are not providing proper follow-up’- P07*.

*‘The palliative care nurses will visit the patients*,* who are unable to get out of bed; I will accompany them for assistance. They will visit once a week or twice.’- ASHA worker-1*.

*‘There are two to three stroke patients here in our settlement*,* palliative care services are provided to them once a month’- ASHA worker-2*.

*The visiting palliative care team only includes nurses and doctors. Other professionals are not visiting the settlement. -Observation*.

### Major theme 9: Health-related issues

The prevalence of chronic diseases is relatively elevated in tribal communities now. Participants reported the conditions of cancer and stroke patients within their community. The tribal people are not receiving consistent attention and follow-up from healthcare professionals.

*‘My grandfather had jaundice*,* which caused him to eat everything without realizing he had the illness. His death was brought on by a delayed diagnosis.’- P08*.

An ASHA worker indicated that the primary cause of mouth and throat cancer among patients in the settlement is the uncontrolled consumption of tobacco and alcohol. All stroke and cancer patients in the settlement had a history of substance use.

*‘One person has been diagnosed with oral cancer in this settlement*,* that is mainly because of the uncontrolled tobacco use’-ASHA worker-1*.

*“Patients with cancers of the mouth*,* throat*,* and stomach are there in some settlements” -AHSA worker-1*.

*“5 Stroke patients are there because of tobacco use”-ASHA worker-1*.

Alterations in lifestyle and poor dietary habits are contributing to more diseases within tribes.

*“Many of the patients have problems with their blood pressure*,* which can lead to stroke. All of this was brought on by an unhealthful diet.”-ASHA worker 2*.

*There is a lifestyle change happening among tribes from traditional to modern- observation.*

## Discussion

The population of scheduled tribes (STs) constitutes a marginalised community that exists in relative social isolation, exhibiting inferior health indices in comparison to similar non-tribal populations [28]. In India, the tribal population is estimated to be 90 million, which accounts for approximately 8.6% of the total population and in Kerala they are 1.45% of the state’s total population [19]. The socioeconomic status and welfare of tribal peoples are transforming, similar to that of other globalised communities [29]. In India, there are numerous healthcare programs designed just for tribal people, and palliative care programs are also a major part of it. Kerala occupies a distinct status from other Indian states [17]. Additionally, Kerala State implemented numerous developmental initiatives in palliative care. The analysis and the selected themes derived from the study suggest that there are still gaps in the case of palliative care among tribal people.

Tribes are recognised for their ancient healing practices. The healing process varied according to the cultural ideas adhered to [30]. The predominant healthcare challenges of the tribes are associated with their culture, habits, and poverty [31]. Their strong beliefs and stakeholders’ limited understanding of their practices and languages constitute significant barriers to the effective provision of healthcare services [32]. Tribes are transitioning from traditional to modern lifestyles these days. This rapid urbanization and lifestyle change increased the prevalence of non-communicable diseases among tribes [19, 33]. This indicates an immediate necessity for culturally sensitive palliative care among tribes. Establishing a hybrid healthcare approach that integrates both traditional and modern medicine can provide excellent palliative care. Healthcare providers interacting with the tribes must be knowledgeable about their traditions and beliefs. There is an immediate need for training programs for professionals engaging with native populations. Tribal people began to progressively utilize the advantages of the government’s accessible programs. However, they are not getting proper attention and follow-up healthcare services for their diseases.

Access to healthcare should be equitable for every individual in our nation. The accessibility of healthcare services designated for tribes remains inadequate. Most of the tribal settlements are located in remote areas of Kerala where the accessibility and transportation services are inadequate [34]. The doctors and nurses are not able to reach the patient immediately in case of an emergency. The Kerala model of palliative care specifically addresses sessions focused on the palliative care of particularly vulnerable individuals. The revised palliative care policy in 2019 specifically states the importance of actively reaching palliative care for ‘care-compromising groups’ including tribes [3]. However, in 2024, the tribes continue to grapple with their own healthcare challenges. Tribes are unable to access available healthcare services in a timely way. The accessible services are inadequately reaching them [35]. The increased prevalence of non-communicable diseases indicates the need for palliative care among the tribal people. These settlements should encompass not only the physical conditions but also the psychological and social aspects. There is an urgent need for counselling services among tribes that are care-focused rather than cure-focused [36]. Psychosocial difficulties are predominantly observed in tribal populations, primarily associated with substance use. They reported an increase in mental health issues among tribes like stress, anxiety suicidal ideation [18, 37]. Most chronic illnesses arise because of uncontrolled substance use [38]. It is essential to educate them and enable them to lead an improved life by sustaining their psycho-social aspects [39, 40]. 

A limited number of tribal settlements in the state of Kerala receive palliative care [36]. Still, there is a high need for palliative care in every settlement [41]. Enhancing awareness of health, illness, and preventive measures is the initial step necessary to elevate the health status of tribes [19]. Lack of awareness is a primary factor contributing to their inadequate health-seeking behaviour, resulting in a lack of timely care [14]. The majority are unaware of the healthcare facilities accessible in their community. Numerous patients who are unable to get out of bed in the tribal hamlet receive insufficient attention and care for their illnesses. It is essential to inform and educate them about palliative care and the services it provides [42]. Palliative care can provide holistic care usually by a multidisciplinary team-based care for people suffering from chronic illnesses [43]. The consistent provision of palliative care services can have a positive impact on their lives. It is the responsibility of every ASHA worker in the settlement to educate individuals about their rights and policies, as well as the patient’s disease and prognosis [33]. Establishing a network of volunteer services specifically for tribal areas is essential to facilitate the adoption and acceptance of palliative care [42]. 

The needs and lifestyle of the rural community differ significantly from those of the urban population [37], so delivering palliative care in rural regions is far more challenging than in urban areas, necessitating additional work for its implementation. We have to start at the foundational level to properly execute tribal palliative care. There should be an increased number of non-governmental organisations (NGOs) dedicated solely to providing palliative care for tribal populations. Collaboration between governmental and non-governmental organisations can facilitate the effective implementation of palliative care [44, 45]. A group of trained healthcare workers impact the attitude of tribes, it will help to build acceptance and trust among them [46]. In addition to physical care, there is a significant need for psychological and social care. Numerous social concerns persist among them, although they continue to struggle with these challenges. Effective home care services provided by the entire palliative care team within their respective communities can systematically improve health promotion and prevention in tribal populations. Enhanced implementation necessitates skilled promoters and ASHA workers to advocate for the rights of tribal communities because they can connect the tribes to society. The healthcare workers’ personal attributes and approach will be significant when working in an ethnic community [46]. It is important to provide culturally respectful and responsive care to tribal people in all settlements [47]. Culturally safe health care influences the perception and pursuit of health services while improving the acceptability and approachability of the health care system within the community [30, 48]. 

### Implication

Providing palliative care for tribal people is as crucial as it is for non-tribal people. Nonetheless, significant gaps persist in addressing the healthcare needs of the tribes. Resolving these identified challenges is crucial for effective implementation. The study shows that the culture and beliefs of tribal people significantly influence their health practices. Developing a hybrid healthcare approach that integrates both traditional and modern medications exclusively for tribes is essential. The approach of healthcare professionals significantly contributes to establishing trust within the tribes. Healthcare professionals must receive adequate training about the culture and traditions of the tribal group with which they engage. Unreported non-communicable diseases exist among tribes in Kerala; these patients require enough attention for their physical, psychological, and social well-being. The study revealed that the tribes are also struggling with psychological and societal challenges. A psychologist and a social worker from the palliative care team should collaborate with the medical staff to address their mental health concerns. Health awareness programs should be implemented in tribal settlements to enhance their health-seeking behaviour. Increased numbers of skilled ASHA workers and promoters are necessary to involve the specific community. Implementing active home-based palliative care services in the tribal settlement is essential to mitigate accessibility-related issues. There is a critical need for culturally competent and informed healthcare policy specifically designed for tribal populations. Supportive policies should evolve together with palliative care policies. Further exploration of palliative care among tribes is essential; further research is required to address existing gaps.

### Limitation

A limited number of participants are included in this study since it is a case study. There may be the chance of more additional factors that researchers are unaware of. This study only included three tribal communities of the Paniya tribe in Kerala. The limited generalisability of the findings is another limitation of the study. The findings can be different if we consider other tribes from other parts of India.

## Conclusion

The health system is experienced by many tribal people as rigid, narrowly focused, and even prejudiced. Palliative care services available to the tribal people are still in the Infancy stage. Lack of awareness, accessibility, financial issues, cultural beliefs and practices, fear and communication barriers and insufficient workforce are the challenges of implementing palliative care among tribal communities. A culturally sensitive hybrid form of palliative care is essential for the tribes, incorporating both traditional and modern medicine. The study reportedly found that a greater initiative like education coupled with health awareness are the determining metrics of their wellness. Palliative care is not only physical but also psychological, social, and spiritual. The proactive effort from the government and other healthcare professionals has been vital for achieving palliative care needs. It is essential to implement and make sure all the programs for tribal health are getting for all the tribal peoples.

## Data Availability

No datasets were generated or analysed during the current study.

## Abbreviations

LMICs: Low and Middle-Income Countries
WHO: World Health Organisation
STs: Scheduled Tribes
ASHA: Accredited Social Health Activist
MGNREGA: Mahatma Gandhi National Rural Employment Guarantee Act
PHC: Public Health Centres
NGOs: Non-Governmental Organisations
